# The ethical aspects of integrating sentiment and emotion analysis in chatbots for depression intervention

**DOI:** 10.3389/fpsyt.2024.1462083

**Published:** 2024-11-14

**Authors:** Kerstin Denecke, Elia Gabarron

**Affiliations:** ^1^ AI for Health, Institute Patient-centered Digital Health, Bern University of Applied Sciences, Biel, Switzerland; ^2^ Department of Education, ICT and Learning, Østfold University College, Halden, Norway; ^3^ Norwegian Centre for E-health Research, University Hospital of North Norway, Tromsø, Norway

**Keywords:** depression, mental health, emotion, sentiment, emotion analysis, chatbot, conversational agent, ethics

## Abstract

**Introduction:**

Digital health interventions specifically those realized as chatbots are increasingly available for mental health. They include technologies based on artificial intelligence that assess user’s sentiment and emotions for the purpose of responding in an empathetic way, or for treatment purposes, e.g. for analyzing the expressed emotions and suggesting interventions.

**Methods:**

In this paper, we study the ethical dimensions of integrating these technologies in chatbots for depression intervention using the digital ethics canvas and the DTx Risk Assessment Canvas.

**Results:**

As result, we identified some specific risks associated with the integration of sentiment and emotion analysis methods into these systems related to the difficulty to recognize correctly the expressed sentiment or emotion from statements of individuals with depressive symptoms and the appropriate system reaction including risk detection. Depending on the realization of the sentiment or emotion analysis, which might be dictionary-based or machine-learning based, additional risks occur from biased training data or misinterpretations.

**Discussion:**

While technology decisions during system development can be made carefully depending on the use case, other ethical risks cannot be prevented on a technical level, but by carefully integrating such chatbots into the care process allowing for supervision by health professionals. We conclude that a careful reflection is needed when integrating sentiment and emotion analysis into chatbots for depression intervention. Balancing risk factors is key to leveraging technology in mental health in a way that enhances, rather than diminishes, user autonomy and agency.

## 1 Introduction

The integration of artificial intelligence (AI) into mental health interventions, particularly in the treatment of depression, represents a significant advance in therapeutic practice in recent years (1). AI-powered chatbots are increasingly being used to provide cost-effective, scalable and accessible mental health services (2). These systems interact with users via text or voice, simulating human-human interaction paradigms. In terms of efficacy, first results reveal that “chatbot-delivered psychotherapy can be adopted in health care institutions as an alternative treatment for depression and anxiety” (3).

In the current landscape, chatbots for depression intervention are primarily used to provide immediate support, psychoeducation and cognitive behavioral therapy (CBT) (4, 5). Some chatbots have been proven to reduce depression and anxiety short-course interventions (6). Further, there is a trend to assess the effectiveness of non-specialized intelligent agents such as ChatGPT (7), Siri, or Cortana in answering questions related to mental health or even support in mental health problems (8). Recently, researchers started to compare ChatGPT with competencies of health professionals for diagnosing and treating patients with depressions (9).

A recent trend is the application of large language models (LLM) to realize natural language processing tasks. An LLM is a machine learning model that encodes complex patterns of language use derived from vast quantities of input texts (10). While some studies indicate that LLMs show promise in providing clinically accurate or good-quality responses (9, 11), other studies point to significant inconsistencies in models’ ability to provide mental health support or advice (12, 13) and their insufficient capacity to manage mental health risk scenarios safely (14). In this paper, the focus is on chatbots designed for depression intervention. However, assessing the ethical risks associated with using non-specialized systems for depression intervention is also highly relevant, especially in the current hype surrounding ChatGPT. Specifically, we are focusing on the ethical dimensions of integrating sentiment and emotion analysis into chatbots for depression intervention.

### 1.1 Approaches to sentiment and emotion analysis

Sentiment analysis is a method that assesses whether an author’s or user’s viewpoint toward a topic, product, or service is positive, negative, or neutral (15, 16). It can be considered at different levels: sentence level, document level and aspect level. The latter is most challenging since various aspects in a sentence may have different polarities and the aspects have to be identified together with related information on them.

Emotion detection goes beyond sentiment analysis by identifying specific human emotions (e.g., anger, joy, sadness). It helps determining an individual’s emotional/mental state precisely (17). Depending on the underlying emotion model, the emotions to be distinguished differ. Broadly, there are two groups of emotion models: Within the dimensional emotion model, emotions are represented based on three parameters: valence, arousal and power. In the categorical model, emotions are defined discretely (e.g. anger, sadness, fear) (15).

The analysis of sentiments or emotions can be realized using a lexicon-based approach, a machine learning-based approach, a deep learning-based approach, a transfer learning or hybrid approach (15). The different types of methods require different resources for their realization, e.g. word dictionaries or training data. An overview on these types of methods is provided as **Supplementary Material**.

There are different lexicons available for sentiment and emotion analysis that have been not specifically developed for the mental health context. Datasets for training originate often from social media. A comprehensive overview of resources for sentiment and emotion analysis in the healthcare domain is provided by Denecke (16). It can be recognized, that most resources are neither specifically from the mental health domain nor from the field of depression. Standard sentiment analysis models are typically trained on datasets that do not adequately represent individuals with mental health conditions. This can lead to biases and inaccuracies when these models are applied to this demographic. Only in 2023, some large language models were published specifically fine-tuned for the mental health domain (18, 19).

### 1.2 Use of sentiment and emotion analysis in mental health chatbots

The application of sentiment and emotion analysis within chatbots allows these systems to detect subtle cues in users’ text or speech that indicate emotional states (20). By analyzing these signals, chatbots can adapt their interactions in real time, providing responses that are empathetically aligned with the user’s emotional needs (21). Devaram’s paper emphasizes the crucial role of empathetic chatbots in mental health care, highlighting their capacity to analyze and respond to users’ emotional states (22). Sentiment or emotion analysis integrated into chatbots for depression could help in providing tailor-made treatments to patients. Extracted opinions or emotions can be used by therapeutic chatbots to suggest an appropriate treatment or to react accordingly (22).

Experiments studying the difference in perceived empathy with ChatGPT compared to neurologists have been conducted; their results indicated that ChatGPT may be superior to neurologists in providing empathy (23). Furthermore, text written by clinicians in collaboration with a chatbot are being perceived as more empathetic than humans (24). These and similar research raises concerns on ethical aspects and safety related to the use of sentiment and emotion analysis in chatbots for mental health in general and for depression in particular (16). Key issues include the accuracy of emotion recognition algorithms, the privacy and confidentiality of sensitive emotional data, and the potential consequences of misinterpreting users’ emotions. Detected emotions can be misused for non-medical purposes including profiling or to manipulate individuals (16). When applying machine learning algorithms to patient data for realizing medical sentiment analysis, multiple instances of biases can be induced which might impact on the clinical decision making.

There is some research available on ethical risks of emotion analysis technology without specific focus on the mental health domain (25). Wang et al. aggregated ethical considerations of using ChatGPT in healthcare (26). Coghlan et al. studied ethical issues with using chatbots in mental health (27). They based their research on the five key ethics principles non-maleficience, beneficience, respect for autonomy, justice, and explicability.

Mohammad (28) discussed several ethical concerns regarding the use of word–emotion association lexicons. One key point is that sentiment lexicons primarily capture implicit emotions—words are linked to sentiments or emotions, but this does not necessarily reflect their true meaning. Different socio-cultural groups interpret words in varied ways; when a lexicon consolidates a specific cultural perspective, it reinforces that interpretation, potentially leading to misleading impressions.

Straw (29) explored the ethical implications of emotion mining in medicine, particularly in the context of the ethical principles of beneficence (promoting the welfare of individuals) and non-maleficence (avoiding harm to individuals). These principles bring into focus critical questions of responsibility and accountability. For instance, the question if a sentiment analysis algorithm fails to detect a suicide risk, who bears the responsibility for the algorithm’s mistake. She also raises concerns related to the impact on equity and fairness. Emotion mining systems may perpetuate biases present in their training data, potentially leading to unfair or discriminatory outcomes for certain demographic groups. Emotion mining in medicine involves analyzing sensitive personal data, raising significant privacy concerns. There are risks of data breaches or misuse of intimate mental health information collected through these systems (29).

Skorburg and Friesen (30) found that using emotions recorded in clinical notes and their use for prediction without patient consent could be interpreted as privacy violation as this might reveal information, patients were not willing to disclose. Although these aspects might also be relevant for sentiment and emotion analysis in chatbots for depression intervention, none of the existing work specifically studied the ethical aspects for this application. Our work differs from the existing research by specifically considering the integration of sentiment and emotion analysis within chatbots for depression intervention. Expressing emotions and reacting empathetically is key of human interaction. Simulating empathy by chatbots or analyzing sentiments and emotions by technology comes along with ethical questions in particular in a mental health settings where systems interact with vulnerable users.

### 1.3 Contributions of this work

A key to reducing concerns regarding specific technology such as sentiment or emotion analysis in chatbots for depression intervention is to systematically assess possible risks. In light of these considerations, the aim of this study is to systematically capture and analyze the risks including ethical risks associated with the integration of sentiment and emotion analysis into chatbots for depression intervention. Given the vulnerability of individuals suffering from depressions and the critical nature of therapeutic interventions, this research aims to assess the potential harms these technologies may pose to users. By identifying and addressing these ethical concerns, the research aims to contribute to the responsible development and use of artificial intelligence in mental health care, ensuring that these innovations truly benefit those they aim to serve.

This paper presents an overview of the ethical aspects and risks associated with integrating sentiment and emotion analysis into a chatbot for depression. The main contributions are:

- a comprehensive assessment of potential risks associated with sentiment and emotion analysis within chatbots for depression by applying the DTx Risk Assessment Canvas (31),
- a thorough evaluation of the ethical risks by applying the Digital Ethics Canvas (32),
- demonstration of the feasibility of using these methods for assessing the risk of technologies to develop mitigation strategies, and
- mitigation strategies for the identified risks.

## 2 Methods

In this study, we focused on chatbots aimed at improving depression specifically. General mental health chatbots were not included unless they directly targeted depressive symptoms. Additionally, we are focusing on purely text-based chatbots for depression intervention, i.e. those that analyze emotions and sentiments expressed in written natural language and thereby exclude systems analyzing face and voice of users.

In this paper, depression interventions refer to chatbots specifically designed to target the symptoms of clinical depression, as reported in the supporting literature. These chatbots are typically grounded in evidence-based therapeutic approaches, such as Cognitive Behavioral Therapy (CBT), and aim to reduce the core symptoms of depression (i.e., persistent sadness, loss of interest, fatigue, and cognitive difficulties among others). Regarding the evaluation of these chatbots, researchers have typically employed a range of outcome measures to track users’ progress, including self-reported symptoms and engagement metrics.

We focused specifically on text-based chatbots and depression interventions because text-based chatbots are a new digital form of intervention that allow users to engage at their own pace, asynchronously and non-intrusively. This type of “interaction” can reduce the pressure some users face with real-time interactions, such as voice or video chats. Text-based chatbot interventions are easily accessible across devices, and require fewer technological resources. Text-based chatbots, as other text-based platforms can also provide a sense of anonymity, which has been shown to increase openness and self-disclosure, particularly in sensitive contexts like mental health. We recognize the potential value in expanding chatbot interventions to other modalities (e.g. voice user interface, avatars) and conditions, and this is something we plan to explore in future work. However, for the scope of this study, we focused on text-based interventions for depression due to their current practicality, and accessibility.

We focused on depression as it is one of the most prevalent mental health conditions globally (33) and often co-occurs with other mental health disorders, such as anxiety, Attention deficit hyperactivity disorder or trauma-related conditions. Effective depression interventions can have a significant impact on overall mental health, as alleviating depressive symptoms can also reduce symptoms of co-morbid conditions. While we acknowledge the importance of addressing a range of mental health issues, we chose to focus on depression due to its widespread impact and the substantial evidence supporting the effectiveness of text-based chatbot interventions for depressive symptoms.

We applied the Digital Ethics Canvas (32), and the DTx Risk Assessment Canvas (31) for collecting ethical and other risks aspects to be considered when integrating sentiment and emotion analysis into chatbots for depression intervention. The tools are described in the following.

### 2.1 Digital Ethics Canvas

The Digital Ethics Canvas (32) is a visual tool for assessing ethical risks from six ethical perspectives specific to the digital domain: beneficence, non-maleficence, privacy, fairness, sustainability and empowerment. By means of guiding questions, researchers and developers are supported in conducting a benefit-risk analysis of the technical solution. Depending on whether the canvas is used in the design or development phase or at use time, mitigation strategies can either be artifact-oriented or context-oriented. That is, the strategies could include changing the technological artifact (e.g., avoid collecting personal data that is not needed to reduce a privacy risk) or changing the usage context (e.g., ask users to provide a nickname rather than their actual name).

In our assessment, we decided to drop the perspective “Sustainability” that is foreseen in the Digital Ethics Canvas since this depends from the approach chosen to realize sentiment or emotion analysis within the chatbot. In the context of chatbots, sustainability refers to the long-term viability and impact of their use over time, including their impact on the environment, as well as on promoting healthy social and economic growth Kotlarsky et al. (34). For example, a chatbot might involve an assessment of the energy efficiency of the algorithm used for emotion analysis, or an ethical handling of the data. In this work, we are not considering a specific approach to sentiment or emotion analysis, but the technology for analyzing emotions and sentiments as such. Therefore, judging the sustainability is not possible.

### 2.2 DTx Risk Assessment Canvas

The potential unintended consequences or risks of using sentiment and emotion analysis in chatbots for depression intervention were assessed using the DTx (Digital Therapeutics) Risk Assessment Canvas (31). Designed for researchers, developers and practitioners, the DTx Risk Assessment Canvas allows to reflect on the potential negative consequences of a digital health solution. It is organized into 15 thematic blocks divided into three groups:

- DTx: Problem, purpose, technology aspects impacting on outcome, clinical evidence, privacy, underlying clinical model;
- Users of the DTx: Individuals using the DTx, behavior of the user, relations of the user;
- Impact of the DTx: Problematic use, relations to other interventions, contraindications, undesired impact, expected outcome, risks and limitations.

For each thematic block, a set of guiding questions is provided, which can form the basis for collecting information on the block (31).

### 2.3 Assessment procedure

**Figure 1:**
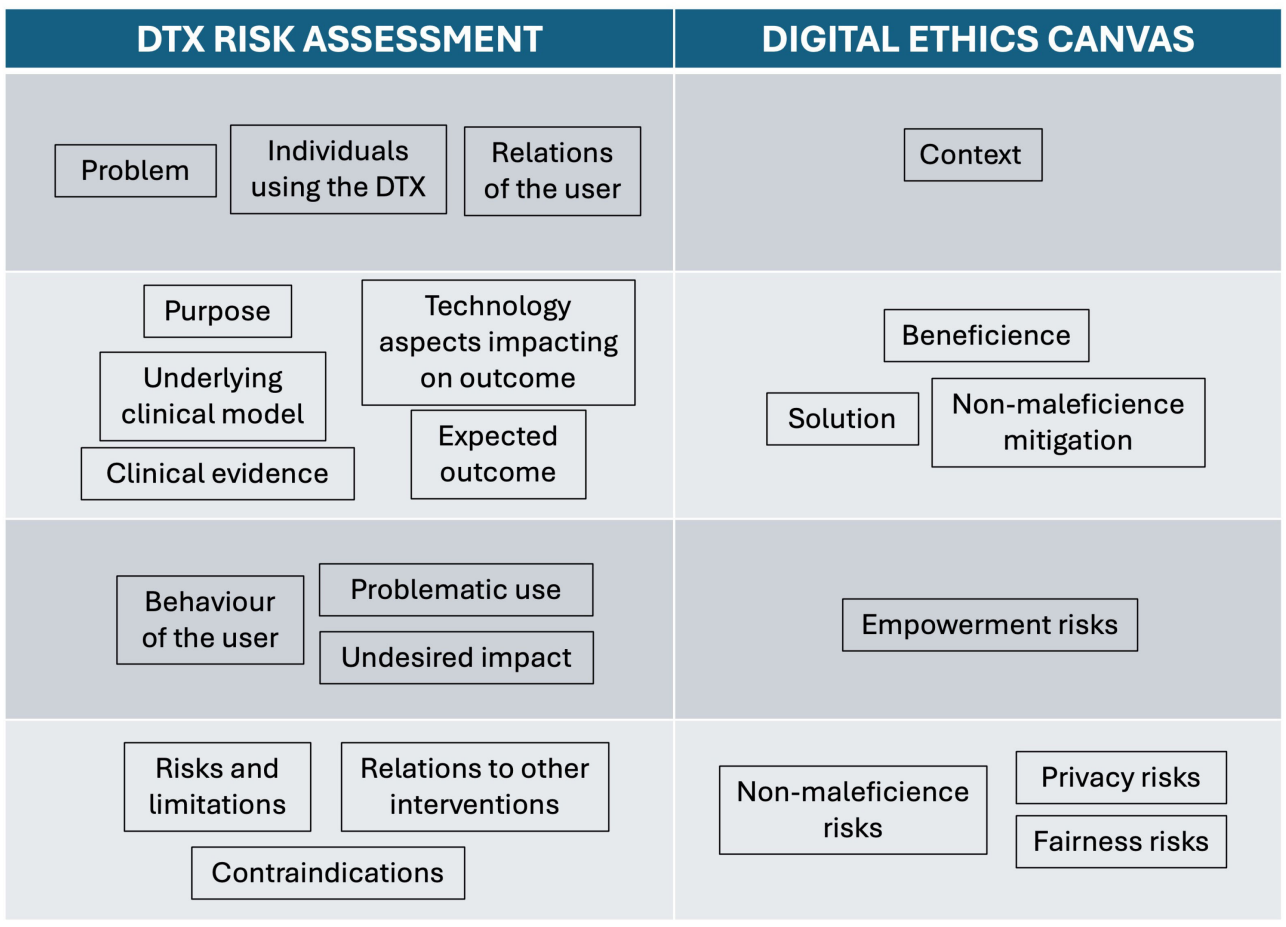
Grouping of thematic blocks from the DTx risk assessment canvas and the digital ethics canvas.

The two authors considered sentiment and emotion analysis integrated into chatbots for depression intervention and reflected on the guiding questions for each thematic block provided by the Digital Ethics Canvas and the DTx Risk Assessment Canvas. Thoughts were collected individually in a Miro board. In an online meeting, the different aspects were discussed, descriptions were concretized and redundant aspects were removed. Reflexive thematic analysis was used to group the thematic blocks from both canvases under similar concepts, as shown in **Figure 1**. It can be seen that the information was grouped into four groups. Then, we searched for concrete examples in literature to support our reflections. For this, we used the literature included into a previously conducted review on chatbots for depression intervention, which was carried out following the PRISMA guidelines (Denecke et al., 2024)^1^. This literature review was conducted on January 8th 2024 and considered the libraries PubMed, ACM Digital Library, IEEExplore, PsychInfo, and CINAHL. We used keywords related to chatbots (chatbot, conversational agent, intelligence agent, virtual assistant) and keywords related to depression (depression, Depressive disorder, dysthymia, affective disorder, dysthymic disorder) for the search. 215 references were found; after duplicate removal and screening of title and abstract, 41 articles remained for full-text review. Additional 18 papers were rejected in that step, resulting in 23 articles included in the review. They refer to 15 chatbots for depression intervention.

The reflections were based on the authors’ experiences in chatbots in mental health (35) and on their experiences in the development (36–39) and analysis (40–42) of chatbots in healthcare. One author has a background in psychology while the other author has a background in computer science and health informatics.

## 3 Results

In this section, we summarize the results grouped in the way that resulted from the reflexive thematic analysis. In section 4, we discuss possible mitigation strategies for the identified ethical and other risks. We first describe the information on the context, user and problem associated with chatbots for depression intervention in general with specific focus on sentiment and emotion analysis. Second, we summarize the purpose of these chatbots, technology aspects related to the outcome, and expected outcome of chatbots for depression intervention and specifically sentiment and emotion analysis. Third, we consider the user behavior and undesired impact followed by the risks and limitations of the technology.

### 3.1 Context, problem, individuals using DTx and relations of the user

Chatbots designed for depression interventions are typically used in two main contexts: either as tools for self-management or self-therapy, or as part of a therapeutic setting. In these scenarios, users may interact with the chatbot as a complement to working with a therapist or as an alternative to traditional therapy methods (43). Chatbots for depression intervention are designed to deliver self-help opportunities and in this way, they aim to mitigate real world problems such as the shortage of mental health staff, the time gap between therapy sessions, and fears of stigmatization. The target users of this technology include individuals diagnosed with clinical depression, but also those exhibiting depressive symptoms without a formal diagnosis, or individuals with anxiety or other disorders with depressive symptoms. Chatbots for depression interventions are designed to serve a diverse range of users across all genders and age groups, from children to younger and older adults - ideally tailored to one specific target group. Additionally, relatives and friends can also utilize the chatbot to support their loved ones.

### 3.2 Purpose, underlying clinical model, clinical evidence, technology aspects impacting on outcome, expected outcome, beneficience, solution

The purpose of chatbots for intervention in depression is to deliver therapy aimed at reducing depressive symptoms. They may also deliver only psychoeducational content or administer some form of co-regulation which may improve depression symptoms but are not necessarily directed therapy. To achieve this, the chatbots may include various therapeutic techniques (44–47), such as training, enablement, goal setting, self-assessment, monitoring symptoms, analyzing responses to treatment or intervention, cognitive restructuring, coping strategies, emotion regulation, environmental restructuring suggestions, psychoeducation, encouraging activities, or incentivization, among others. The chatbot should obtain a clear overview of the user’s emotional state and be capable of identifying emergencies, such as suicide risk or other situations of emotional risks. The underlying clinical model of these chatbots commonly reported in the literature is Cognitive Behavioral Therapy (CBT) as therapeutic model (37, 45, 48–52).

For realizing sentiment and emotion analysis, sentiment models (three classes, 5-item scale), and emotion models (45) are considered when designing the algorithms to be integrated in the chatbot. On the clinical evidence, while CBT efficacy has been extensively studied in therapeutic settings, there is very limited evidence of CBT delivered through chatbots (53). Nevertheless some research has shown that the use of chatbots - including those incorporating sentiment or emotion analysis - lead to a significant improvement of depression symptoms (43, 45, 52, 54, 55). Furthermore, our assessment highlights that there is no evidence on the impact of emotion analysis and empathy in chatbots on users. The efficacy of sentiment or emotion analysis integrated into these systems seems to have not yet been studied independently from other involved therapeutic techniques, making it difficult to judge the specific contribution of sentiment and emotion analysis alone.

The expected outcome of interacting with the chatbot is a reduction or elimination of depressive symptoms and related symptoms, prevention of relapse, and improved user knowledge about depression and coping strategies. In terms of technological aspects impacting outcomes, it is important to consider that emotions can be indirectly expressed by users through descriptions of situations and that a single statement can contain multiple sentiments and emotions, making an automatic analysis difficult. Chatbots for depression are mostly text-based and use various methods for emotion analysis, such as rule-based (43, 56), machine learning (37, 44, 45, 57, 58), deep learning (51), and transfer learning.

On the benefits of using chatbots for depression, our assessment indicates that it is important to highlight their anonymity, accessibility (easy access, ease of use, and availability 24/7) (44), their potential to benefit vulnerable residential populations (59) and the emotional support they can provide. Regarding the solution, the canvas evaluation suggests that chatbots can offer guidance and information, help users feel respected and less judged, allow for the monitoring of sentiments and emotions to recognize possible patterns, reduce feelings of isolation, and encourage openness about emotions without fear of criticism or misunderstandings. Additionally, the sentiment and emotion analysis methods integrated can help in tailoring content according to the user’s emotional state and identify crises by analyzing expressed emotions. This together with empathy shown towards the user can lead to a closer bond of trust between the user and the technology (47), increasing its acceptance.

### 3.3 Behavior of the user, problematic use, undesired impact, and empowerment risks

Regarding user behavior, we have identified that the user can ask the chatbot for tips to improve depressive symptoms, but also for other issues unrelated to depression (e.g., groceries), or even for disclosing crimes (27). User interactions can follow heterogeneous usage patterns (60), and high dropout rates have been reported (49, 54). On the problematic use, it is anticipated that the user may display self-harm behaviors that would require appropriate reaction. They may also provide only short statements open to interpretation leading to failure in the sentiment and emotion analysis. Users might not disclose their true emotions and sentiments to the chatbot, hiding or masking them. The adherence to the chatbot intervention can be low which is specifically problematic when this intervention is aimed at replacing face-to-face interaction with a therapist. Problematic use could also include the user preferring interaction with the bot due to more predictable emotional reactions, developing an addiction to the chatbot, or not interacting with it at all. On the undesired impact, we presume that there can be an over-reliance on the tool’s capacity and the risk of developing a false sense of security and trust in human-like chatbots because of their built-in empathy and emotional analysis, perceiving them as capable of providing the same level of care as human therapists (61). This may lead to users disclosing sensitive information and relying on the chatbot for depression intervention, which the chatbot may not be equipped to deal with effectively. On the other hand, increased dependency on the chatbot caused by the adaptation of the system to user preferences could increase isolation. As exemplified, a chatbot would most probably be always polite and will not be designed to show negative emotions towards its user. This could be perceived in a positive way by the user, becoming unwilling (or unable) to interact with humans and deal with the different behaviors of humans.

Empowerment risks may involve the user’s lack of interest in the chatbot due to depressive symptoms or non-interaction. Additional risks of empowerment could be associated with the lack of transparency in the communication of privacy policies (62), difficulties in understanding sentiment and emotion analysis technologies, the lack of transparency about the capabilities and limitations of emotion analysis, the lack of explicit consent for emotion analysis, and the user potentially developing a dependency on the empathy or emotional support provided by the chatbot.

### 3.4 Risks and limitations, relations to other interventions, contraindications, non-maleficience risks, privacy risks, and fairness risks

Risks and limitations of using chatbots for depression intervention and specifically integrating sentiment and emotion analysis identified by the canvas assessment include the potential for providing conflicting, incomplete, inaccurate, or incorrect information, lacking empathy, and missing context, especially when only analyzing text without sources of information such as voice patterns or facial expressions. Users could misinterpret the chatbot’s formulations, feel uncomfortable interacting with a machine, or prefer human interaction. Regarding contraindications and relation to other interventions, we have inferred that chatbots might be contraindicated for individuals at high risk of suicide and those in therapy settings that conflict with chatbot recommendations. Non-maleficence risks may include the chatbot misunderstanding the user, especially with irony, which can negatively affect the user’s mood. Chatbots may lack empathy, provide misinformation, or even give information on self-harm. Emotion analysis could be biased due to training data or dictionaries, and misunderstandings can arise from users’ impaired capacity to express emotions. Chatbots might overlook suicide and self-harm behavior, and the quality of emotion analysis might be low.

For many people with depression who feel isolated, interacting with chatbots might increase feelings of isolation, especially if the chatbot pretends to be a good friend who never expresses negative emotions. This can result in a loss of skills in handling criticism or misunderstandings related to emotions and sentiments. Privacy risks noted in the canvas assessment include the collection of personal and sensitive data, enabling emotional profiling, lack of encryption for conversations, and vulnerability to hacking, which could allow third-party access to data. Fairness risks could include ignoring cultural or educational differences in expressing emotions (63), missing context leading to unfair suggestions, and models trained on specific datasets not generalizing well to diverse populations with depression among others, resulting in inaccurate analysis. People who cannot express their emotions well might not achieve good results with the chatbot (64).

## 4 Discussion

### 4.1 Main findings

Despite their potential benefits regarding anonymity and accessibility (27), chatbots for depression intervention must navigate significant risks. The main findings from the canvas assessments highlight several significant general risks associated with the use of chatbots for depression intervention (e.g. user discomfort with machine interaction, missed detection of critical situations), but also risks that are specifically associated with the integration of sentiment and emotion analysis into these systems (e.g. misunderstandings of irony, bias in sentiment analysis). Primarily, these risks include the potential for chatbots to provide conflicting or inaccurate information due to low-quality emotion analysis due to technical limitations or limitations in contextual understanding which would be necessary for an appropriate interpretation of the expressed emotion. User misinterpretation of chatbot responses and discomfort with machine interaction were identified as risks, with particular concerns for those at high risk of suicide, for whom chatbots may not provide adequate support. The analysis also identified issues with bias in emotion recognition models due to unrepresentative training data, leading to inaccurate emotional assessments, particularly for users from different cultural or educational backgrounds.

In addition, the findings point to the risk of increased isolation due to over-reliance on chatbots. The risk of over-reliance could be considered for any chatbot, but it may increase when the chatbot becomes more human-like due to the integration of emotion and sentiment analysis. Research showed users who perceive chatbots as more human-like and conscious also report higher perceived social health benefits from interacting with them, suggesting a relationship between human-likeness and user engagement, where increased anthropomorphism may lead to stronger emotional attachments (65). There is a risk of user dependency on chatbots, highlighting the need for balance and integration with human support systems to ensure comprehensive mental health care. Further, privacy concerns around data collection are rather general risks of chatbots. However, the emotional profiling is specifically associated with the integration of sentiment and emotion analysis, increasing the risk of misusing this information. **Table 1** summarizes the main risks and possible mitigation strategies that will be described in more detail in the following.

**Table 1:** Summary of risks associated with sentiment and emotion analysis in chatbots for depression and mitigation strategies.

Risk	Mitigation strategy
**Missing context**	Integrate additional data on patient context, e.g. from electronic health record to enhance context understanding and avoid misinterpretations of emotions and sentiments due to missing context.
**Misunderstanding of irony and complex emotions**	Enhance natural language processing models to better detect and understand irony and complex emotional expressions; provide users with instructions on how to communicate more clearly with the chatbot.
**Bias in sentiment or emotion analysis due to training data**	Use diverse and representative domain-specific datasets for training analysis models; continuously monitor and adjust algorithms to minimize bias.
**Low quality sentiment or emotion analysis**	Continuously improve sentiment or emotion analysis algorithms through research and development and learning from user interactions; validate analysis outputs with human mental health professionals; train chatbot on diverse and empathetic dialogue datasets, ideally from mental health.
**Fairness risks ignoring cultural and educational differences**	Train models on datasets that include diverse cultural and educational backgrounds; develop customizable settings to better cater to individual user differences.
**Privacy risks from data collection and profiling**	Implement strong data encryption and secure storage practices; ensure transparent communication about data usage and obtain explicit user consent.
**User dependency on chatbot**	Educate users about the supplementary role of chatbots in mental health care; inform about limitations; implement features that periodically encourage users to seek human interaction and support.
**Increased isolation from over-reliance on chatbots**	Encourage regular interactions with human therapist; design the chatbot to promote real-world social interactions and activities.
**Missed detection of critical situations**	Implement robust emergency detection and response protocols; ensure clear communication about the limitations of the chatbot in handling crisis situations.
**User discomfort with machine interaction**	Offer options to transition to human therapists when needed.

The risks aggregated in this table are described in sections 3.3 and 3.4.

### 4.2 Recommendations for mitigation of identified risks

Several risks could be addressed by embedding the chatbot use in a care setting. For example, mitigating user discomfort with interacting with a chatbot - although or because it is human-like - could be realized by offering the option to transit to a human therapist out of the chatbot dialogue. Additionally to mitigate potential harm (non-maleficence), it is suggested to employ human supervision (27). Chatbot developers should ensure that the chatbot provides sufficient benefits to compensate for potential harms (27). Given its criticality, we describe aspects related to risk assessment within chatbots for depression intervention in a separate subsection.

Depending on the implementation of the sentiment and emotion analysis, the associated risks differ. Lexicon-based approaches require dictionaries. Although in these dictionaries meanings of words are fixed allowing for a reproducible analysis, the general purpose sentiment and emotion dictionaries may ignore the expressions used by depressed individuals. Constant updates are needed to incorporate new linguistic patterns. Rule-based systems struggle with context and may misinterpret the sentiment if the user’s input does not match the predefined patterns, leading to inappropriate or harmful advice.

In case the sentiment and emotion analysis relies upon training data, the data might originate from other domains, may include biases and stereotypes (66, 67). Biased or unrepresentative data can lead to inaccurate sentiment analysis and inappropriate responses. Machine learning models are highly dependent on the quality and quantity of training data. It is thus important to use diverse and representative domain-specific datasets for training the sentiment and emotion analysis algorithms. Frequent system updates, learning from user interactions and the use of datasets from the mental health domain are also recommended. Careful assembly of the training data and human assessment of the results are essential.

The use of pre-trained models raises ethical concerns about the consent and privacy of the original data. Transferring this knowledge to sensitive areas such as mental health requires careful consideration. Further, the effectiveness of transferring general or different domain sentiment or emotion analysis models to the mental health domain learning can be inconsistent, depending on how well the pre-trained model’s knowledge applies to the new context, potentially leading to unpredictable or unreliable chatbot behavior.

There is evidence that empathy is an important element of any therapeutic relationship and that patients have moderately better outcomes in psychotherapy when they perceive therapists as understanding them (68). However, it is still unclear in which situations empathy may be particularly valuable or conversely contraindicated (68). The concrete effects of sentiment and emotion analysis integrated into chatbots for depression intervention still have to be assessed.

Providing users with information on the chatbot’s limitations and any associated risks is necessary (64) to avoid over-reliance and user dependency. Transparency about the technology involved in chatbots for depression intervention, including its limitations, is crucial. Developers should ensure mechanisms to get informed consent of users not only to the general use of the chatbot, but on the analysis of emotions and sentiments and the respective data collection and data use (27). This is because of the sensitive nature of emotion and sentiment information. In principle, users should be provided with detailed information about how the sentiment and emotion algorithms work, what they are used for, which data will be used, and the potential benefits, limitations, and risks associated with their use (69). Only when knowing how a system will process the data, users can decide what they are willing to disclose. Developers must rigorously test and improve the sentiment and emotion analysis methods to minimize errors and prioritize user safety and equity.

### 4.3 Addressing safety-critical situations

In our risk assessment, we have highlighted that chatbots for depression intervention should obtain a clear overview of the user’s emotional state and be capable of identifying emergencies, such as suicide risk. However, chatbots might overlook signs of suicide and self-harm behavior, making them potentially contraindicated for individuals at high risk of suicide. Chatbots for depression intervention should include features for emergency identification and maintain transparency about limitations to ensure safety and effectiveness. Ethical concerns also include the potential for chatbots to inadvertently encourage self-harm, as highlighted by cases where chatbots suggested suicide, emphasizing the need for rigorous guidelines and continuous monitoring. Specifically, it has been documented an incident where ChatGPT-3, when interacting with a simulated patient, suggested committing suicide (70). More alarmingly, it has also been reported a tragic real-world case where a user followed through with suicide after being encouraged by the chatbot Eliza (71). These cases underscore the urgent need for rigorous ethical guidelines and supervision in the development of chatbots. Ensuring that these digital tools do not exacerbate users’ mental health issues requires continuous monitoring, human oversight, and the implementation of robust safety protocols to prevent harmful interactions.

These examples demonstrate that chatbots such as ChatGPT, which are not specifically designed for mental health purposes, when misused as depression intervention tools, they can provide inconsistent or inappropriate responses that may exacerbate rather than alleviate users’ mental health conditions, and in this way risk patient safety. General-purpose chatbots lack the nuanced understanding and training that mental health professionals have, which could lead to misdiagnoses or harmful advice. Conversely, chatbots designed specifically for depression intervention also carry ethical risks. They may inadvertently reinforce negative thought patterns or fail to recognize severe symptoms that require immediate professional intervention. Relying on these chatbots could discourage individuals from seeking help from human professionals, leading to delayed treatment of serious conditions. In addition, both types of chatbots raise privacy concerns, as sensitive personal data could be mishandled or exploited, further compromising users’ well-being. For chatbots that are not specifically designed for depression intervention, this risk might be even higher. Therefore, while these technologies hold promise, the potential for harm requires strong ethical guidelines and robust oversight to ensure their safe and effective use. Health systems must ensure through regulation that chatbots maintain patient safety and equity in care, requiring robust experimental work for diverse patient populations (69).

### 4.4 Limitations of this work

This study acknowledges several limitations that must be considered. The digital risks and ethics analyses were conducted by only two individuals. This limited scope may have led to the dismissal of potential risks and the failure to identify new risks that could emerge as technologies evolve. To mitigate this, future assessments should be conducted with a panel of experts and chatbot users to ensure a comprehensive evaluation. We were focusing on purely text-based chatbots. There might be different and additional risks associated with sentiment and emotion analysis when analyzing voice or facial expressions of the user (22). Each chatbot is unique and the individual differences in people with depression present significant variability too. Therefore, each case should undergo specific risks and ethics assessments tailored to the particular chatbot and individual circumstances. We recommend to apply the two tools that were used in this work when developing chatbots for depression intervention. They will help to reflect on the risks and integrate mitigation strategies right from the beginning. Furthermore, although there is a growing body of literature on the development and testing of chatbots for depression interventions, there is a gap in the discussion regarding the risks and ethical implications of such applications. Our study underscores the necessity for more in-depth exploration and dialogue on these critical aspects to ensure the responsible and safe use of chatbots in mental health care.

Therapeutic interventions involve structured, and evidence-based approaches aimed at treating disorders; while interventions offering support or psycho-educational approaches only, although being a type of intervention and providing useful support and encouragement, are typically not considered as therapeutic interventions. In our assessment, we considered publications on chatbots designed as interventions for depression and did not differentiate between therapeutic and non-therapeutic chatbots, as our goal was to provide a broad overview on the ethical dimensions of integrating sentiment and emotion analysis in chatbots for depression intervention. We identified literature undermining our reflections from a recently conducted review on chatbots for depression (Denecke et al., 2024)^1^. This procedure may have overlooked aspects. However, since the review was following the PRISMA guidelines, we are confident that it provides an unbiased view on the current landscape of chatbots for depression.

## 5 Conclusions

In this paper, we assessed the risks associated with sentiment and emotion analysis in chatbots for depression intervention using two assessment tools. We identified several risks that can be grouped into risks related to the capabilities of a chatbot in general, risks associated to the quality of sentiment and emotion analysis, risks associated with the data collection and processing and risks associated with the user behavior. By describing these risks together with possible mitigation strategies, our study aims to raise awareness of the ethical dimensions inherent in decision-making processes during the development of mental health chatbots, and to stimulate discussion within the field. Ethical considerations surrounding the integration of sentiment and emotion analysis into these chatbots have not been sufficiently addressed by the research community. This oversight occurs despite the unique challenges and risks to patient safety posed by these technologies. Consequently, our work is dedicated to elucidating the significance and consequences of ethical and risk factors the integration of sentiment and emotion analysis in chatbots for depression.

In summary, while chatbots with integrated sentiment and emotion analysis can enhance user autonomy and agency by providing personalized, accessible, and confidential support, it is crucial to address potential drawbacks such as dependency, privacy concerns, and the risk of misinterpretation. Balancing these factors is key to leveraging technology in mental health in a way that enhances, rather than diminishes, user autonomy and agency. Further research is needed to disentangle these effects and evaluate how much of the observed benefits are directly attributable to sentiment and emotion analysis versus other components of the chatbot’s design and functionality.

## Data Availability

The original contributions presented in the study are included in the article/**Supplementary Material**. Further inquiries can be directed to the corresponding author.

## Footnotes

1 Denecke K, Rivera Romero O, Wynn R, Gabarron E. Persuasive chatbot-based interventions for depression: A list of recommendations for improving reporting standards. Front Psychiatry. (2024). Under review.
