# Supplementary material for: The ethical aspects of integrating sentiment and emotion analysis in chatbots for depression intervention
Source: Front Psychiatry. 2024 Nov 14;15:1462083. doi: 10.3389/fpsyt.2024.1462083 (PMC11602467; doi:10.3389/fpsyt.2024.1462083)
Supplement: Supplementary file 1 [file DataSheet1.pdf]

# Supplementary Material

## 1 SUPPLEMENTARY TABLES

### 1.1 Sentiment and emotion analysis approaches

The following table provides a brief overview on the approaches to sentiment and emotion analysis, their required resources and approaches to analyze sentiment or emotion.

| | **Lexicon-based** | **Machine learning-based** | **Deep learning based** | **Transfer learning** |
|---|---|---|---|---|
| **Required resources** | Word dictionary with words associated with the polarity or emotion category. Dictionary can be manually built or extracted from a corpus. | Training data set (annotated or not annotated) | No or pre-trained model from the domain (e.g. BERT, GPT-4) | Pre-trained model from a related domain |
| **Approach to sentiment or emotion analysis** | Decision is made based on the sum or mean of the sentiment values or emotions expressed by single words | Feature extraction and classification using Support Vector Machines, Naive Bayes, Decision tree etc. | Models extract features themselves. Convolutional Neural Networks, Long Short-Term Memory, Large Language Model | Fine-tuning of the model for the domain |

**Table S1.** Sentiment and emotion analysis approaches
